# Polymorphisms in the p63 and p73 genes are associated with ovarian cancer risk and clinicopathological variables

**DOI:** 10.1186/1756-9966-31-89

**Published:** 2012-10-24

**Authors:** Xiao Guan, Ning Zhang, Yongshuo Yin, Beihua Kong, Qifeng Yang, Zhiyan Han, Xingsheng Yang

**Affiliations:** 1 Department of Obstetrics and Gynecology, Qilu Hospital, Shandong University, 107#, Wenhua Xi Road, Jinan, P.R. China; 2 Department of Breast Surgery, Qilu Hospital, Shandong University, 107#, Wenhua Xi Road, Jinan, P.R. China; 3 Department of Surgical Oncology, Dongguan People's Hospital of Guangdong Province, 3#, New Guchong Road, Dongguan, P.R. China; 4 School of Public Health, Shandong University, 44#, Wenhua Xi Road, Jinan, P.R. China

**Keywords:** Polymorphism, Single nucleotide polymorphisms, Ovarian cancer, p73, p63

## Abstract

**Objective:**

p73 and p63 are two structural and functional homologs of p53, and their biological functions in cancer progression have attracted attention due to the presence of variants generated by genetic polymorphisms. Recently, three single nucleotide polymorphisms (SNPs) in the p63 and p73 genes have been associated with female reproduction. In the present study, we aimed to evaluate the relationship between these SNPs and ovarian cancer susceptibility and clinical pathology.

**Methods:**

We genotyped the p63 (rs873330 [Genbank, refSNP ID] T > C [T: original base, C: mutant base]) and p73 (rs4648551 G > A and rs6695978 G > A) SNPs in ovarian cancers and healthy controls and analyzed the distributions of genotype frequencies to evaluate the association of the genotypes with the risk of ovarian cancer and the clinicopathological characteristics. Logistic regression models were applied in statistical analyses.

**Results:**

Our research revealed that p73 rs6695978 G > A was significantly associated with ovarian cancer patients. Women with the A allele were at increased risk of ovarian cancer compared to carriers of the G allele (OR = 1.55; 95% CI:1.07–2.19; *P* = 0.003). Meanwhile, the at-risk A allele was positively related with the occurrence of mucinous ovarian cancer (OR = 3.48; 95% CI:1.15-6.83; *P* = 0.001), low degree of differentiation (OR = 1.87; 95% CI:1.03-3.47; *P* = 0.003), lymph node metastasis (OR = 1.69; 95% CI: 1.14-2.75; *P* = 0.010) and estrogen receptor positive (OR = 2.72; 95% CI: 1.38-4.81; *P* = 0.002). However, we were unable to find any associations of the polymorphisms in another two SNPs (rs4648551 G > A, rs873330 T > C) with ovarian cancer risk and clinicopathological parameters.

**Conclusions:**

The p73 rs6695978 G > A polymorphism will serve as a modifier of ovarian cancer susceptibility and prognosis. Further investigations with large sample sizes and of the mechanistic relevance of p73 polymorphism will be warranted

## Introduction

Ovarian cancer is a serious threat to the lives and health of women around the world. The incidence rate of ovarian cancer, which varies among ethnic groups and geographic regions, has increased dramatically in recent years. In China, there are more than 192,000 women diagnosed with ovarian cancer, with approximately 114,000 deaths annually. Ovarian cancer has become the second most common malignancy in Chinese women. Despite major advances made in its treatment, ovarian cancer continues to have the highest fatality of all gynecologic malignancies [1]. Approximately 70% of all ovarian cancers were diagnosed at an advanced stage due to the difficulty of early diagnosis and widespread intra-abdominal metastasis. Gene susceptibility has been reported to potentially play a significant role in ovarian carcinogenesis [2]. Therefore, identifying predisposing genes to establish high-risk groups and achieve early diagnosis may be beneficial to improve the survival rate of ovarian cancer.

The process of tumor formation and regulation appears to entail a complex combination of genetic, environmental and lifestyle factors. Complex diseases such as cancer, including ovarian cancer, have been hypothesized to arise due to the effect of many low-risk gene variants that collectively increase disease risk [3]. Single nucleotide polymorphisms (SNPs) are the most common sequence variations in the human genome, and they involve only a single base mutation and can affect coding sequences, splicing and transcription regulation. SNPs can comprehensively reflect genomic hereditary and variation with large quantity, high density, wide distribution and typical representation. Therefore, SNPs may play increasingly important roles in screening for the gene mutations and the susceptibility to oncogenic factors [4].

The p63 and p73 genes belong to the p53 superfamily of transcription factors, which contribute to cell cycle regulation, transactivation and apoptosis in response to DNA damage [5]. Despite sharing a similar structure and function, each p53 family member appears to play a distinct role in tumor suppression and progression. Interestingly, high levels of certain p63 and p73 isoforms have been observed in some tumors, suggesting that these proteins may act as oncogenes rather than classic tumor suppressor proteins [6-9]. Furthermore, p63 and p73 genes regulate ovary functions and female germ cell integrity in humans. The two genes overexpression may play catalytic roles in ovarian epithelial tumor development because both of them can produce synergistic effects on ovarian tissue malignant transformation and enhance the tumor invasion ability. The relatively new Genome-wide association study (GWAS) approach has investigated hundreds of thousands of genetic variants across the whole human genome for associations with cancer [10]. Recently, there has also been mounting evidence that both the p63 and p73 genes play important roles in human cancer, and their biological behaviors in cancer progression have been revisited in light of variants generated by genetic polymorphisms. However, little is known about how the p63 and p73 polymorphisms are involved in ovarian cancer susceptibility and clinical pathology. In particular, three SNPs (rs873330 T > C, rs4648551 G > A, rs6695978 G > A) located in p63 and p73 have been confirmed to have a clear enrichment of specific alleles in infertility and in vitro fertilization (IVF) patients [11]. Infertility, controlled ovarian hyperstimulationmay (COH) may be factors predisposing to ovarian cancer diseases [12]. Infertility therapies utilize products, such as IVF, that alter the hormonal balance and may in theory increase the risk of ovarian tumors. Children born after IVF therapies seem to have a statistically elevated risk of cancer [12,13].

Based on these observations between infertility and ovarian cancer risk, we sought to investigate whether the p63 and p73 polymorphisms could serve as susceptible and/or progressive factors in ovarian cancer. To analyze whether the distributions of their genotype frequencies are associated with clinicopathological characteristics, we performed genotyping analyses of p63 (rs873330 T > C) and p73 (rs4648551 G > A, rs6695978 G > A) in a case–control study of 308 ovarian cancer cases and 324 healthy controls in a Chinese population.

## Materials and methods

### Patients and samples

This study involved 308 patients diagnosed with ovarian cancer in Qilu Hospital (Shandong, China) between January 2008 and September 2011. All ovarian cancer cases were classified and assessed according to the American Joint Committee on Cancer (AJCC) and International Federation of Gynecology and Obstetrics (FIGO) classification, and the pathological types were diagnosed with epithelial ovarian cancer, germ cell tumor, and sex gonad stromal tumor using conventional pathological examination or immunohistochemistry after surgical excision. The histologic subtypes were classified into serous, mucinous, endometrioid and mixed/other roughly due to the detailed ovarian tumor histology data were not available. The exclusion criteria included reported previous cancer history and metastasized cancer from other organs. To illustrate whether the three SNPs in p63 and p73 are susceptible biomarkers, 324 women from a screening program for non-infectious and major diseases conducted from 2009 to 2010 in the same hospital were included as the control group in this study. The matching criterions between the cases and the controls include age, BMI (body mass index), number liveborn, oral contraceptive use, cigarette smoking, ovarian cancer family history. For these two groups, a 1.5 ml whole blood sample was extracted from each participant and stored at −80°C. Written informed consent was signed by each subject, and the study design was approved by the Ethical Committee of Shandong University.

### DNA extraction

Genomic DNA for all subjects was extracted from whole blood using the Qiagen blood kit (Chatsworth, CA, USA) following the manufacturer’s instructions. The DNA concentration and purity of each sample were measured using an ultraviolet spectrophotometer (GE Healthcare, Piscataway, NJ, USA). The genomic DNA samples were marked with a specimen No. and stored at −80°C.

### SNP genotyping analyses

TaqMan allelic discrimination analyses were performed according to Applied Biosystems standard protocols (Applied Biosystems, Carlsbad, CA, USA). The SNPs were as follows: rs4648551 (C_26892242_10), rs6695978 (C_1210727_10), and rs873330 (C_3208788_10) (Applied Biosystems Inc. ABI). Each 10 μl reaction was composed of 1 μl of genomic DNA (100 ng/μl), 5 μl of UMM (TaqMan Genotyping Master Mix, ABI, Part No. 4371355), 0.5 μl of probes (rs4648551/ rs6695978/ rs873330, ABI), and 3.5 μl of DNase-free water. The PCR was performed according to the following amplification protocol: denaturation at 95°C for 10 min, followed by 50 cycles of 92°C for 15 s and 60°C for 1 min and final annealing and extension at 60°C for 30 s. The PCR products were analyzed by the 5’ nuclease assay (TaqMan®) on the Applied Biosystems Prism 7900HT Fast-Real-time PCR system using the StepOne Software Version 2.2 (ABI).

### Statistical analyses

As quality control, the genotype and allele frequencies of rs4648551 G > A, rs6695978 G > A and rs873330 T > C were calculated using a public statistical Web-tool, http://www.oege.org/software/hwe-mr-calc.shtml, for Hardy-Weinberg equilibrium (HWE). A *P* value > 0.05 was considered as not deviating from equilibrium according to population genotype frequencies. Logistic regression models were established to analyze the distributions of the three SNP polymorphisms between the case and control groups and the clinicopathological characteristics of ovarian cancer. *P* values and Odds Ratios (ORs) were adjusted for age, BMI, number liveborn, oral contraceptive use, cigarette smoking, ovarian cancer family history. All statistical tests were considered significant at a level of *P* ≤ 0.05. All statistical analyses in our study were conducted using SPSS Statistics 16.0 (SPSS Inc. Chicago, IL, USA).

## Results

### The genotype distribution satisfied the hardy-Weinberg equilibrium

All ovarian cancer patients and healthy controls were local women in Shandong Province, China. The average age of cases and controls were 52.90 ± 13.26 and 49.89 ± 13.48 years, respectively, and the Student’s t test did not show significant differences between the two groups (*P* = 0.082). Furthermore, we did not find statistically significant differences between the two groups in other matching characteristics except ovarian cancer family history (*P* = 0.003) (Table 1). A chi-squared test was used to determine whether the subjects were in Hardy-Weinberg equilibrium. The distributed genotype frequencies of these three SNPs (rs4648551 G>A, rs6695978 G>A, rs873330 T>C) conformed with Hardy-Weinberg equilibrium in both the case and control groups (Table 1), which demonstrated that the population in this study reached genetic equilibrium with typical group representation.

**Table 1:** Distributions of select variables (covariate data) in the cases and controls and test of the Hardy-Weinberg equilibrium for the SNPs

**Variables**	**Cases, n = 308**	**Controls, n = 324**	***P***
Age, year (mean ± SD)	52.90 ± 13.26	49.89 ± 13.48	0.082
Body mass index, kg/m^2^			0.23
< 23	85 (27.6)	92 (28.4)	
23-29	157 (51.0))	178 (54.9))	
≥ 29	66 (21.4)	54 (16.7)	
Number liveborn, n (%)			0.064
0	19 (6.2)	17 (5.2)	
1-2	227 (73.7)	258 (79.6)	
≥ 3	62 (20.1)	49 (15.1)	
Oral contraceptive use, n (%)			0.49
never	184 (59.7)	201 (62.0)	
1-48 months	55 (17.9)	47 (14.5)	
≥ 48 months	69 (22.4)	76 (23.5)	
Cigarette smoking			0.76
Yes	6 (1.9)	4 (1.2)	
No	302 (98.1)	320 (98.8)	
Ovarian caner family history			0.003^a^
Yes	29 (9.4)	7 (2.2)	
No	279 (90.6)	317 (97.8)	
Hardy-Weinberg equilibrium			> 0.05^b^
rs 4648551	χ2 = 22.3; *P* =0.98	χ2 = 0.05; *P* =0.99	
rs 6695978	χ2 = 0.04; *P* =0.81	χ2 = 10.19; *P* =0.85	
rs 873330	χ2 = 0.16; *P* =0.72	χ2 = 0.10; *P* =0.75	

a. There are no statistically significant differences between the two groups in the select variables (covariate data) except ovarian cancer family history.

b. *P* >0.05 indicate genotype distributed frequencies in the cases and controls conformed with Hardy-Weinberg genetic equilibrium.

### The p73 rs6695978 G > A SNP can enhance susceptibility to ovarian cancer.

This case–control study included 308 ovarian cancer cases and 324 cancer-free controls. The genotype distributions of the p73 (rs4648551 G > A, rs6695978 G > A) and p63 (rs873330 T > C) polymorphisms between the case and control groups are shown in Table 2. We concluded that the frequency of the A allele in p73 rs6695978 G > A was statistically higher in the case group compared with the control group. Women with the A allele were at increased risk of ovarian cancer compared to carriers of the G allele (OR = 1.55; 95% CI: 1.07-2.19; *P* = 0.003). Of interesting, we found that the at-risk A allele in rs6695978 G > A was significantly associated with ovarian cancer in the allele dose–response manner. That is, carrying the GA and AA genotypes may increase ovarian cancer susceptibility by 1.64-fold (95% CI: 1.37-1.95; *P* = 0.004) and 1.81-fold (95% CI: 1.56-2.14; *P* = 0.004) compared with the GG genotype respectively. The data in Table 2 indicated that no associations of p63 rs873330 T > C and p73 rs4648551 G > A with ovarian cancer pathogenesis were found. In summary, we determined that the rs6695978 A allele may be the at-risk allele for ovarian cancer, suggesting that carriers of the A allele may be more susceptible to ovarian cancer among Chinese women.

**Table 2:** Logistic regression analyses on associations between p63 rs873330, p73 rs4648551, rs6695978 and risk of ovarian cancer

**Gene and SNP**	**Genotype of SNP**	**No. of subjects (%)**		**Adjusted**^a^	
		**Controls**	**Cases**	***P***	**OR (95 % CI)**
p63					
rs873330 T > C	TT	182 (56.7)	160 (52.0)	0.142	1.00 (ref)
	TC	118 (36.8)	122 (39.6)		1.15 (0.88-1.52)
	CC	21 (6.5)	26 (8.4)		1.21 (0.78-1.89)
	T allele	482 (75.1)	442 (71.8)		
	C allele	160 (24.9)	174 (28.2)	0.098	1.16 (0.79-1.68)
p73					
rs4648551 G > A	GG	316 (97.5)	296 (96.1)	0.936	1.00 (ref)
	GA	8 (2.5)	10 (3.3)		1.05 (0.91-1.22)
	AA	0 (0.0)	2 (0.6)		
	G allele	640 (98.8)	602 (97.7)		
	A allele	8 (1.2)	14 (2.3)	0.558	1.41 (0.99-1.93)
rs6695978 G > A	GG	240 (74.1)	198 (64.3)	0.004	1.00 (ref)
	GA	73 (22.5)	94 (30.5)		1.64 (1.37-1.95)
	AA	11 (3.4)	16 (5.2)		1.81 (1.56-2.14)
	G allele	553 (85.3)	490 (79.5)		
	A allele	95 (14.7)	126 (20.5)	0.003	1.55 (1.07-2.19)

a. OR and 95% CI represent odds ratios and 95% confidence intervals from logistic regression analysis, adjusted for age, BMI, number liveborn, oral contraceptive use, cigarette smoking, ovarian cancer family history.

All statistical tests were two-sided with a significance level of *P* ≤ 0.05.

### The p73 rs6695978 G > A SNP was positively associated with known clinicopathological variables.

Considering that none of the investigated SNPs except the p73 rs6695978 G > A had shown an association between the case group and the control group, we merely listed the data between the rs6695978 G > A genotype frequencies and the clinicopathological characteristics, including age at diagnosis, tumor histology, degree of differentiation, clinical stage , tumor behavior, lymph node status, estrogen receptor (ER) and progesterone receptor (PR) status (Table 3). The results from the logistic regression models revealed that the A allele was positively associated with the occurrence of mucinous ovarian cancer (OR = 3.48; 95% CI:1.15-6.83; *P* = 0.001), low degree of differentiation (OR = 1.87; 95% CI:1.03-3.47; *P* = 0.003), lymph node metastasis (OR = 1.69; 95% CI: 1.14-2.75; *P* = 0.010) and ER positive (OR = 2.72; 95% CI: 1.38-4.81; *P* = 0.002), which can be used to predict disease prognosis and treatment outcomes. However, our analysis did not show significant associations of the polymorphism with age at diagnosis, clinical stage, tumor behavior and PR status. Therefore, the p73 rs6695978 G > A polymorphism may be a potential indicator of clinical prognosis in Chinese female ovarian cancer patients.

**Table 3:** Relationship between the p73 (rs6695978 G > A) polymorphism and known clinicopathological variables of ovarian cancer

**Clinicopathological Variables**	**All**	**Genotype(%)**		**A allele frequency**	**Adjusted**^**a**^	
		**GG**	**GA+AA**		***P***	**OR (95 % CI)**
Age	308				0.948	
< 52	118	88 (74.6)	30 (25.4)	0.136		1.00 (ref)
≥52	190	146 (76.8)	44 (23.2)	0.137		2.87 (0.93-5.84)
Clinical stage	300				0.474	
I-II	92	69 (75.0)	23 (25.0)	0.131		1.00 (ref)
III-IV	208	158 (76.0)	50 (24.0)	0.142		1.30 (0.89-1.93)
Tumor histology	308				0.003	
Serous	196	150 (76.5)	46 (23.5)	0.128		1.00 (ref)
Mucinous	24	15 (62.5)	9 (37.5)	0.250	0.001	3.48 (1.15-6.83)
Endometrioid	22	17 (77.3)	5 (22.7)	0.114	0.337	2.25 (0.96-4.44)
Mixed/other	66	52 (78.8)	14 (21.2)	0.136	0.597	0.93 (0.76-1.19)
Degree of differentiation	246				0.005	
High	28	22 (78.6)	6 (21.4)	0.107		1.00 (ref)
Medium	82	65 (79.3)	17 (20.7)	0.104	0.827	1.15 (0.86-1.69)
Low	136	98 (72.1)	38 (27.9)	0.162	0.003	1.87 (1.03-3.47)
Tumor behavior	294				0.838	
Borderline	48	37 (77.1)	11 (22.9)	0.125		1.00 (ref)
Invasive	246	191 (77.6)	55 (22.4)	0.122		0.91 (0.79-1.03)
Lymph node status^b^	176				0.010	
Negative	62	50 (80.6)	12 (19.4)	0.105		1.00 (ref)
Positive	114	83 (72.8)	31 (27.2)	0.154		1.69 (1.14-2.75)
ER^c^	183				0.002	
Negative	42	36 (85.7)	6 (14.3)	0.095		1.00 (ref)
Positive	141	100 (70.9)	41 (29.1)	0.163		2.72 (1.38-4.81)
PR^c^	171				0.329	
Negative	66	49 (74.2)	17 (25.8)	0.144		1.00 (ref)
Positive	105	81 (77.1)	24 (22.9)	0.129		1.43 (0.76-2.32)

a Logistic regression model adjusted for age, BMI, number liveborn, oral contraceptive use, cigarette smoking, ovarian cancer family history.

b For advanced ovarian cancer patients, in terms of primary cytoreductive surgery, whether to simultaneously apply pelvic and para-aortic lymph node dissection is controversial. The general consensus that pelvic and para-aortic lymph node dissection does not increase the 5-year survival rate and improve prognosis has been widely accepted. Thus, some patients involved in our study only underwent primary cytoreductive surgery without pelvic and para-aortic lymph node dissection. The data regarding lymph node status in patients were partially missing.

c Unlike breast cancer and endometrial cancer, the significance of ER and PR in the clinical treatment and prognosis of ovarian cancer is also valuable and disputed. Meanwhile, combined with the economic condition of the patients, some cases did not undergo ER and PR immunohistochemical analyses.

All statistical tests were two-sided with a significance level of *P* ≤ 0.05.

## Discussion

Recent studies have revealed that several genetic polymorphisms may play important roles in the pathogenesis of ovarian cancer [14,15], and women who carried the gene mutation (BRCA1 mutation) had an increased risk (by up to 50%) of developing ovarian cancer in a lifetime [16]. In one of the most recent genome-wide association study (GWAS) conducted in ovarian cancer, a strong association with 12 single-nucleotide polymorphisms (SNPs) in the Basonuclin 2 (BNC2) gene was found, where the minor allele of the associated SNPs were protective against disease [17,18]. Moreover, a meta-analysis showed that patients with one of single nuclear polymorphisms vitamin D receptor (VDR), the FokI rs2228570 TT genotype, had a significantly higher risk for developing ovarian cancer as well as prostate, breast, skin, non-Hodgkin lymphoma, and colorectal cancer compared with its CC genotype [19,20]. By seeking susceptibility genes and establishing high-risk populations, early diagnosis may be beneficial to improve ovarian cancer survival.

As tumor candidate genes, p63 and p73 are involved in the regulation of the cell cycle, apoptosis, differentiation and other critical cellular processes. The abnormal expression of the two genes can play catalytic roles in the development of ovarian tumors and achieve synergy in terms of early malignant transformation and enhanced tumor invasion. In recent years, there has been an increased interest in research into the connection between p63 and p73 variants generated by genetic polymorphisms and cancer progression. Meanwhile, several genetic polymorphisms have been implicated in the pathogenesis of ovarian cancer [14-20]. However, little is known about how the p63 and p73 polymorphisms are involved in ovarian cancer susceptibility and clinical pathology. Therefore, we conducted this study to genotype three SNPs in the p63 and p73 genes to determine whether this polymorphism functioned as a modifier of ovarian cancer development.

Prior studies have demonstrated that p63 and p73 were highly expressed in female germ cells during meiotic arrest and play an important role in DNA damage-induced apoptosis in female germ cells [21,22]. Recently, three SNPs (rs873330 T > C, rs4648551 G > A, rs6695978 G > A) located in p63 and p73 were identified, and they appear to be under evolutionary selection pressures using the criteria of Atwal [23,24] and information theory. That study showed a clear enrichment of the SNPs in infertility and IVF patients and revealed that polymorphisms in the human p63 and p73 genes could be involved in reproductive deficits [11,25]. In theory, the factors including non-pregnancy, infertility and application of ovulation induction drugs that lead to continued ovulation can increase the incidence of ovarian cancer [26]. Infertility therapies utilize products, such as IVF, that alter the hormonal balance and may also increase the risk of ovarian tumors [12]. Based on the close relationship between infertility and ovarian cancer susceptibility, we genotyped these SNPs in ovarian cancer patients and normal individuals using a case–control study. Our results indicated that the A allele frequency in p73 rs6695978 G > A was statistically higher in the case group compared with the control group. Women with the A allele were at increased risk of ovarian cancer compared to carriers of the G allele, which also confirmed that this polymorphism may serve as a predictor of ovarian cancer susceptibility.

Ovarian cancers comprise a broad spectrum of malignancies, ranging from serous to mucinous, endometrioid, clear cell and other histologic subtypes. These histotypes have been recently associated with distinct molecular profiles and polymorphisms, supporting the idea that the distinct molecular pathways and genotype may strongly affect germinal histotypes in ovarian cancer [27-30]. As demonstrated in our research, the frequencies of A allele and combined GA + AA genotypes in p73 rs6695978 G > A were statistically elevated in mucinous ovarian cancer compared with other subtypes. In all histologic types, the prognosis of mucinous ovarian cancer is unsatisfactory and prone to metastasis and drug resistance [31], which introduces difficulties and challenges into clinical treatment. The median survival (MS) of mucinous adenocarcinomas did not differ significantly between the groups interpreted as primary or metastatic, but the overall survival (OS) of mucinous adenocarcinomas is significantly less than that for women with serous carcinoma [32]. Mucinous carcinomas are independent predictors of poor prognosis in stage III/IV ovarian cancer [33]. Consequently, our study indicates that the rs6695978 A allele may be more closely related to the occurrence of mucinous ovarian cancer and individuals carrying the AA and GA genotypes may suffer from poorer prognoses.

With respect to the clinical stage, differentiation degree and lymph node status in ovarian cancer, which can guide clinical treatment and outcomes, there is also mounting evidence that p73 protein expression may play a vital role in malignant transformation, clinical stage, differentiation degree and metastasis of ovarian cancer [34]. The lower the differentiation degree, the higher the level of p73 expression. Increasing the expression of the p73 protein in tumor cells may strengthen the suppression of cellular apoptosis; the survivability and malignancy of the tumor are enhanced accordingly. Thus, tumor cells may have an easier time to invade the surrounding tissues and metastasize to the lymph nodes, which shorten the survival of ovarian cancer patients. In this study, we clarified the association of the A allele frequency in the p73 rs6695978 G > A with a low degree of differentiation and lymph node metastasis, which is similar to what was seen in previous studies on the status of p73 expression in ovarian cancer. However, the biological relevance for an association between rs6695978 A allele and ovarian cancer remains unclear. Therefore, we will sought to assess whether there was a necessary link between p73 expression status and the p73 rs6695978 G > A polymorphism in the next step. Further investigation in a larger sample size involved molecular mechanisms are indispensable.

Ovarian cancer is a hormone-dependent malignancy, and estrogen/ progesterone and its receptors may play important roles in the pathogenesis of ovarian cancer. For a long time, progesterone has been considered to be a protective factor for ovarian cancer. Approximately 26% to 49% of ovarian cancers have PR expression [35], and patients with a high expression of PR often have a good prognosis [36]. In contrast, estrogen has been considered as a risk factor for epithelial ovarian cancer. The proliferation of ovarian tissue with estrogenic stimulation and estrogen/hormone replacement therapy (HRT) may possibly increase the risk of ovarian cancer [37,38]. Approximately 61% to 79% of ovarian cancers express the ER [35]. From the pathological standpoint, estrogen and ER expression can accelerate the mitosis of ovarian cancer cells, which rely on inhibiting apoptosis and promoting cell proliferation to participate in the development of tumors. Hence, ER-positive ovarian cancer patients often suffer from a poor prognosis. The data in our research illustrated that ER-positive patients tend to carry the AA and GA genotypes in p73 rs6695978 compared with the GG genotypes. In contrast with ER-negative, the A allele frequency in rs6695978 were also statistically increased in ER-positive patients. There appears to be a potential connection between rs6695978 A allele and bad clinical outcomes.

In conclusion, this is the first study to indicate the p73 rs6695978 G > A A allele as the at-risk allele may enhance susceptibility to ovarian cancer in Chinese women. The individuals with the A allele were at increased risk of ovarian cancer compared to carriers of the G allele, and positively associated with the occurrence of mucinous ovarian cancer, poor differentiation, lymph node metastasis and estrogen receptor status, which all indicate a poor prognosis for ovarian cancer. However, detailed ovarian tumor histology data were not available, and the biological and mechanistic relevances between rs6695978 A allele and ovarian cancer remain unclear. Meanwhile, the process of ovarian cancer development in women is probably mediated by other candidate genotypes and different pathways; this analysis leads to future work in the following directions (a) with large samples and detailed surveys focusing on the functional pattern of this polymorphism (b) examination of p73 expression levels by genotype among the current population. (c) analysis of genotypic interactions with closely-related genes. Further research of this critical gene and those which are biologically related may lead to a better informed biological understanding of ovarian cancers. Substantiating its independent prognostic value for clinical diagnosis and outcome is of great significance. In addition, findings such as these will lead to the development of genetic risk prediction panels for eventual classification of women who may most benefit from targeted surveillance or prevention strategies.

## Abbreviations

SNP: Single nucleotide polymorphisms; GWAS: Genome-Wide Association Study; IVF: In vitro fertilization; AJCC: American Joint Committee on Cancer; FIGO: International Federation of Gynecology and Obstetrics; BMI: Body mass index; ABI: Applied Biosystems Inc.; HWE: Hardy-Weinberg equilibrium; OR: Odds Ratios; MS: Median Survival; OS: Overall Survival; ER: Estrogen receptor; PR: Progesterone receptor; HRT: Hormone replacement therapy.

## Competing interests

All authors declare that we have no financial and non-financial competing interests after the publication of the manuscript.

## Authors' contributions

Xiao Guan carried out the molecular genetic studies inciuding DNA extraction and SNP genotyping analyses, and drafted the manuscript. Ning Zhang participated in analyzing the distribution of genotype frequency. Yongshuo Yin carried out the collection of clinicopathological characteristics. Beihua Kong and Qifeng Yang took part in the design of the study. Zhiyan Han performed the statistical analysis. Xingsheng Yang conceived of the study, and participated in its design and coordination and helped to draft the manuscript. “All authors read and approved the final manuscript”.
